# Supplementary figures and images for: WASH regulates the oxidative stress Nrf2/ARE pathway to inhibit proliferation and promote apoptosis of HeLa cells under the action of Jolkinolide B
Source: PeerJ. 2022 Jul 13;10:e13499. doi: 10.7717/peerj.13499 (PMC9288166; doi:10.7717/peerj.13499)

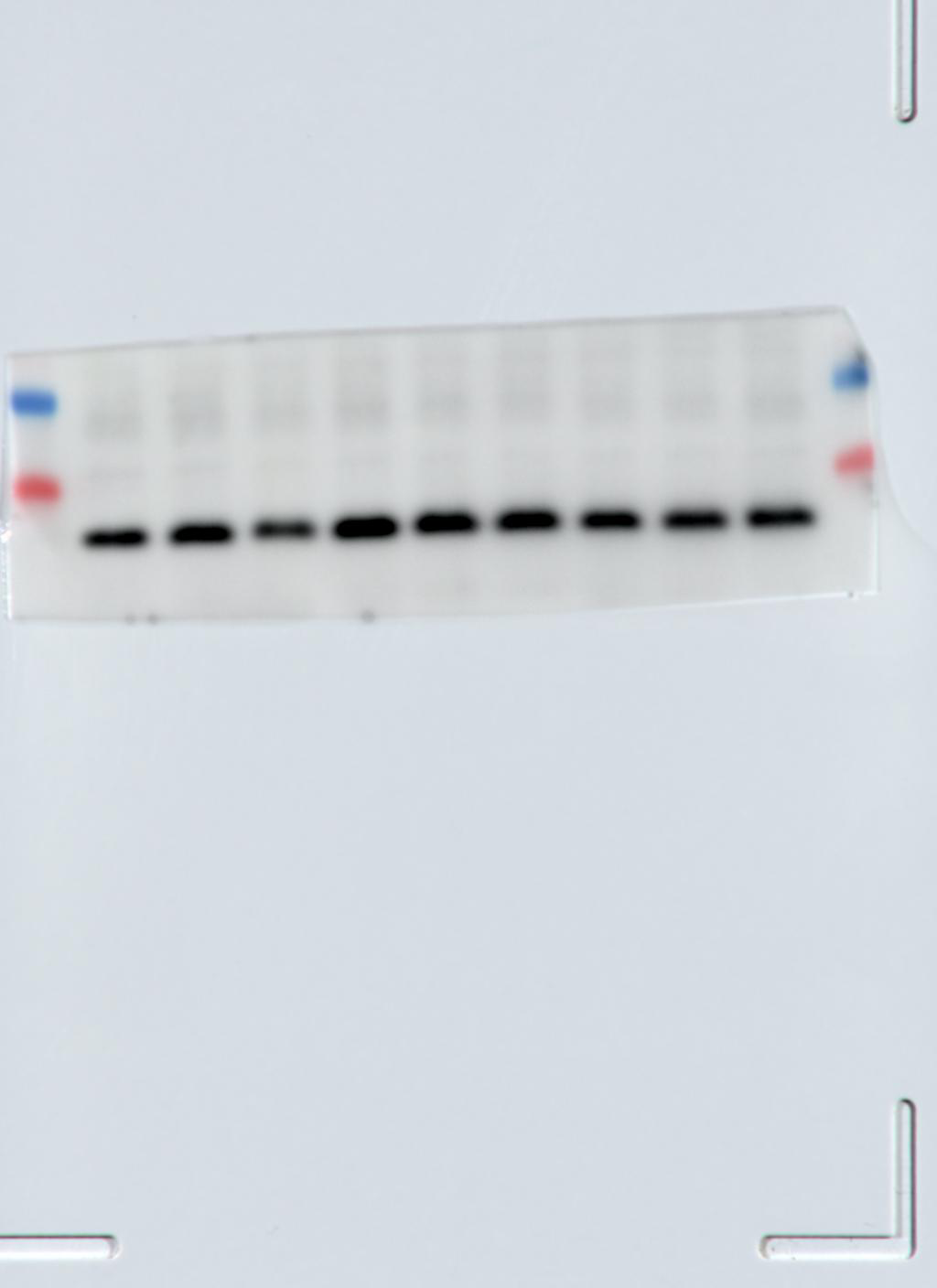

Supplement: Supplemental Information 1 [file peerj-10-13499-s001.zip › WB results of JB treating in hela-shWASH cells/WB results of JB treating in hela-shWASH cells-First/BAX/BAX.png]

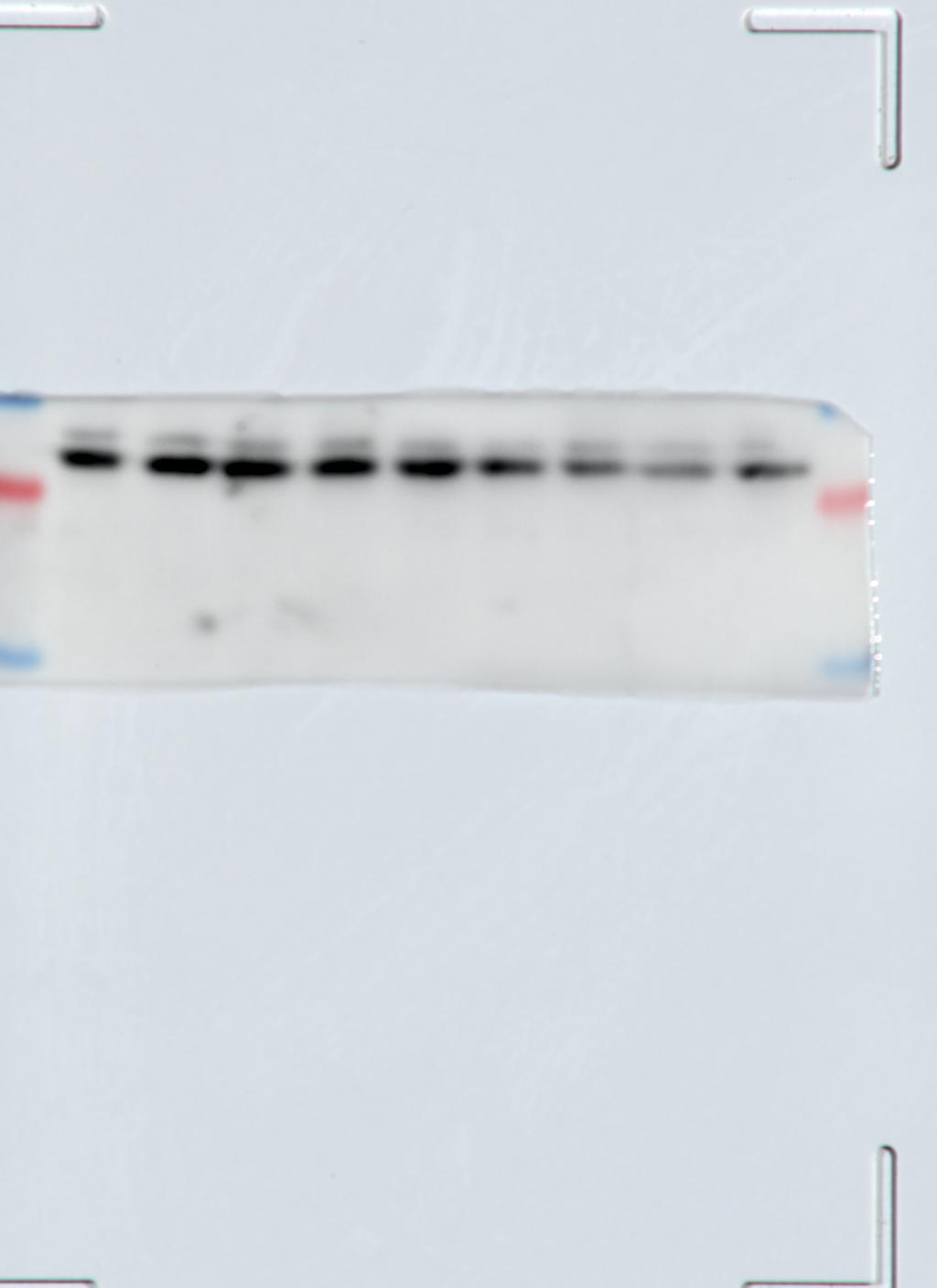

Supplement: Supplemental Information 1 [file peerj-10-13499-s001.zip › WB results of JB treating in hela-shWASH cells/WB results of JB treating in hela-shWASH cells-First/BCL2/BCL2.png]

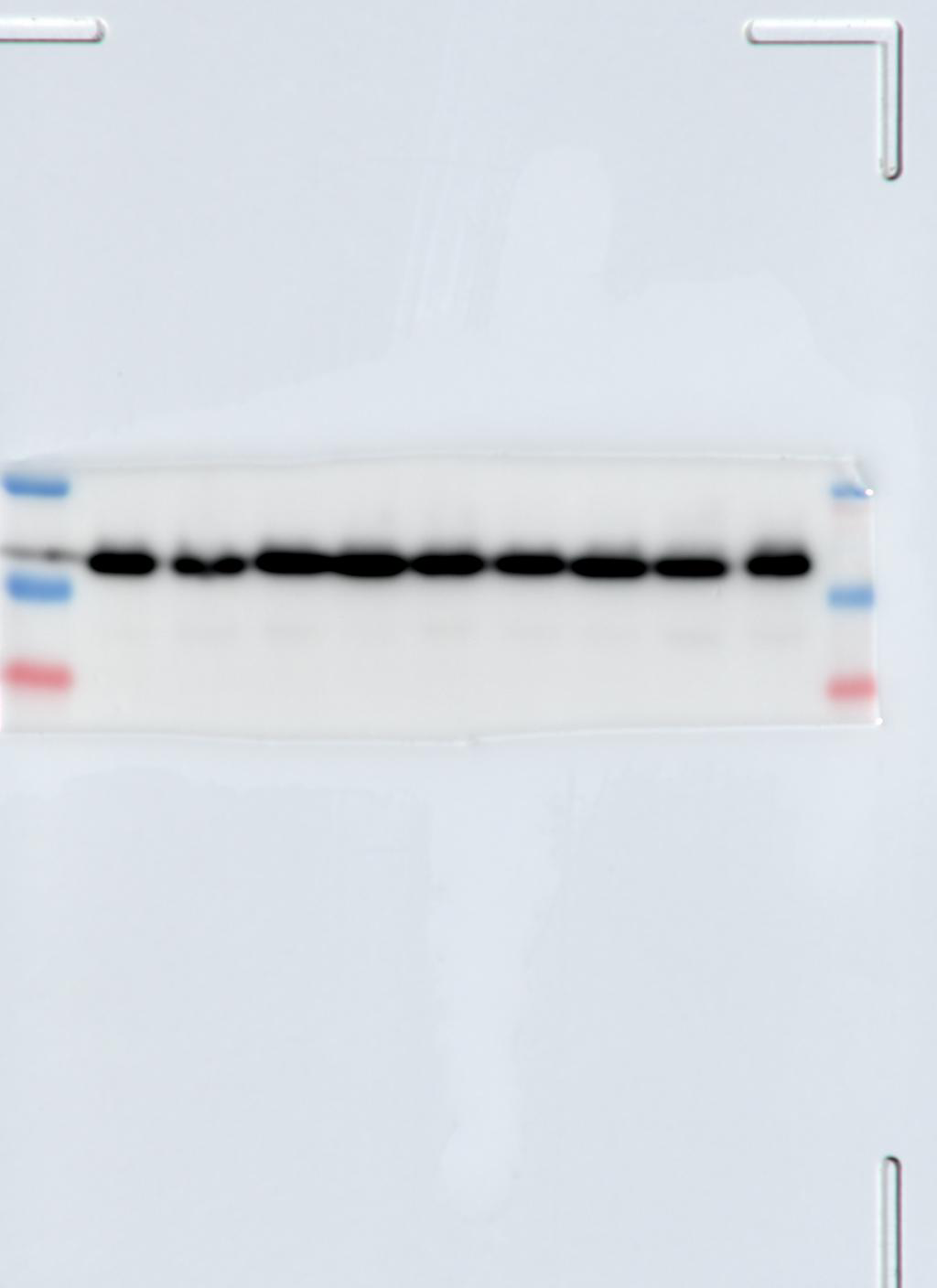

Supplement: Supplemental Information 1 [file peerj-10-13499-s001.zip › WB results of JB treating in hela-shWASH cells/WB results of JB treating in hela-shWASH cells-First/GAPDH/GAPDH.png]

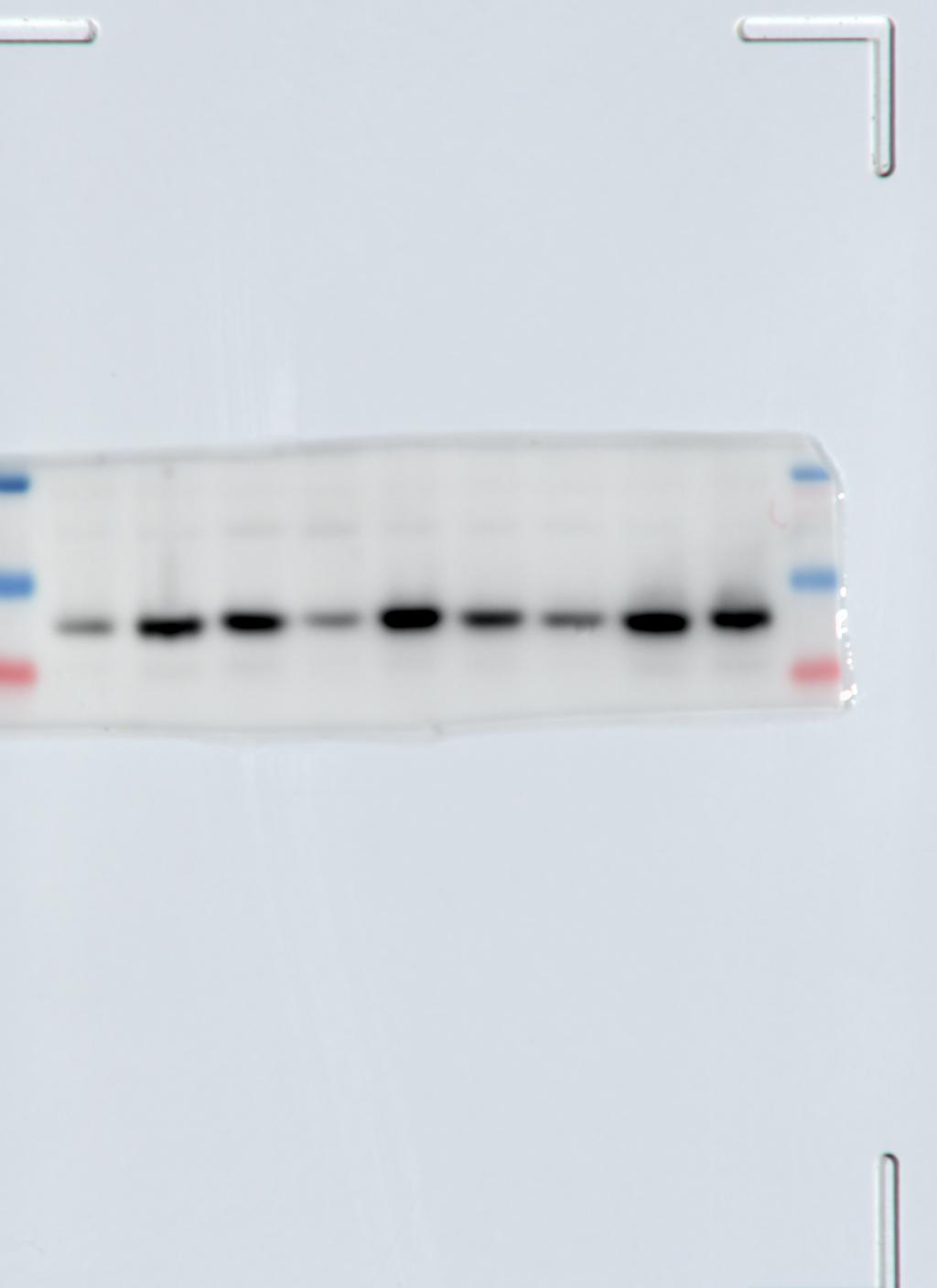

Supplement: Supplemental Information 1 [file peerj-10-13499-s001.zip › WB results of JB treating in hela-shWASH cells/WB results of JB treating in hela-shWASH cells-First/HO-1/HO-1.png]

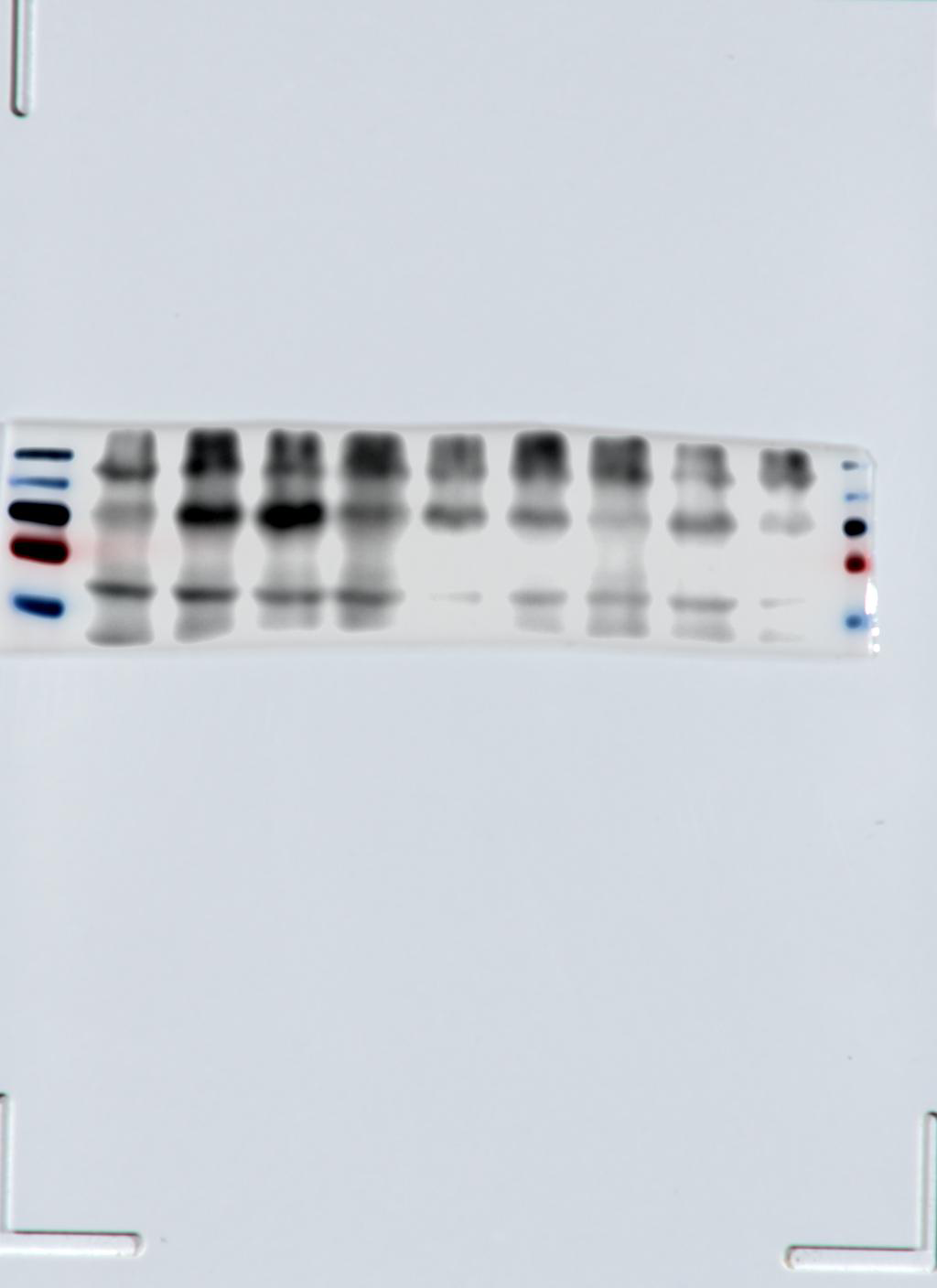

Supplement: Supplemental Information 1 [file peerj-10-13499-s001.zip › WB results of JB treating in hela-shWASH cells/WB results of JB treating in hela-shWASH cells-First/NRF2/NRF2.png]

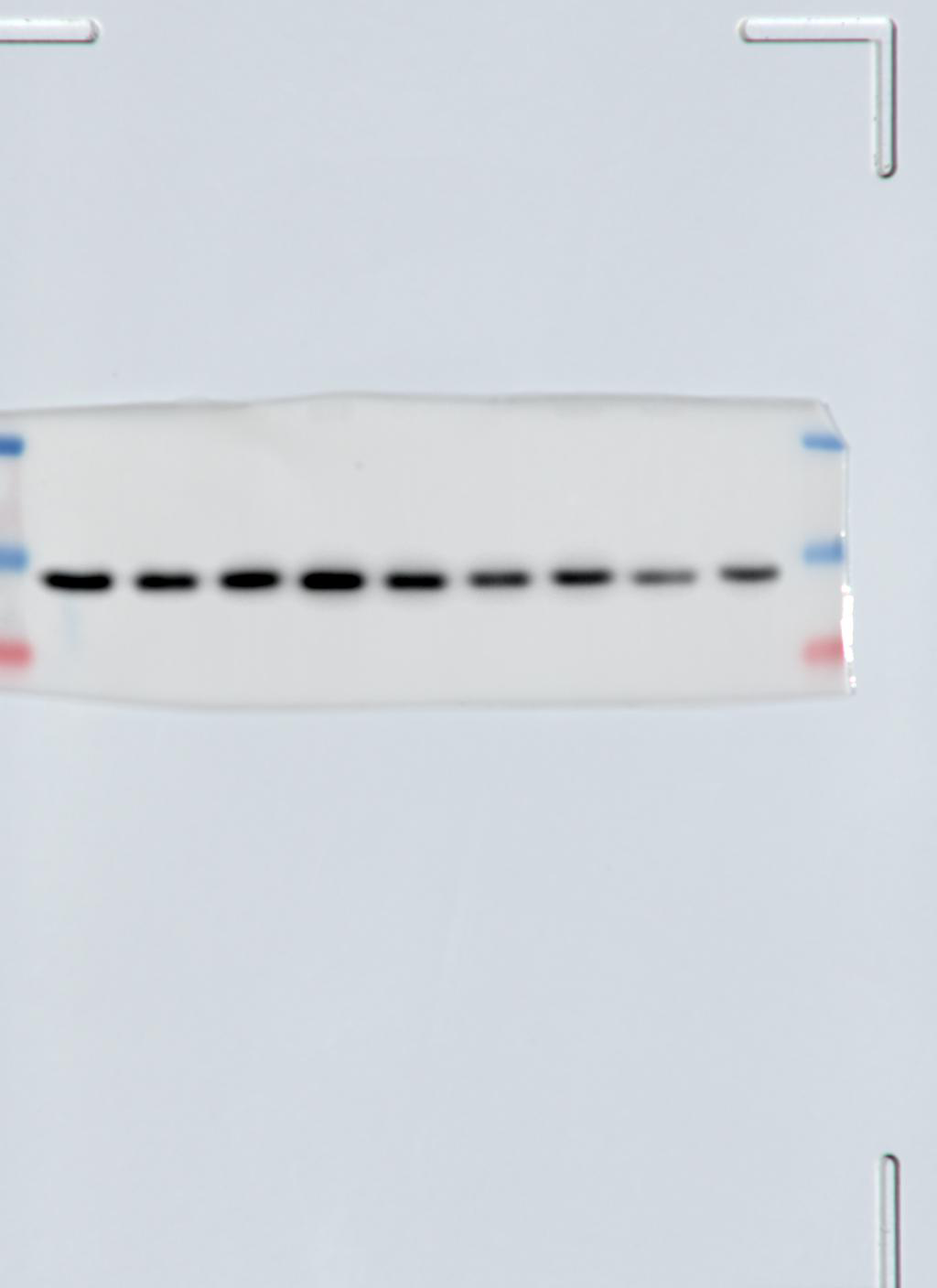

Supplement: Supplemental Information 1 [file peerj-10-13499-s001.zip › WB results of JB treating in hela-shWASH cells/WB results of JB treating in hela-shWASH cells-First/PCNA/PCNA.png]

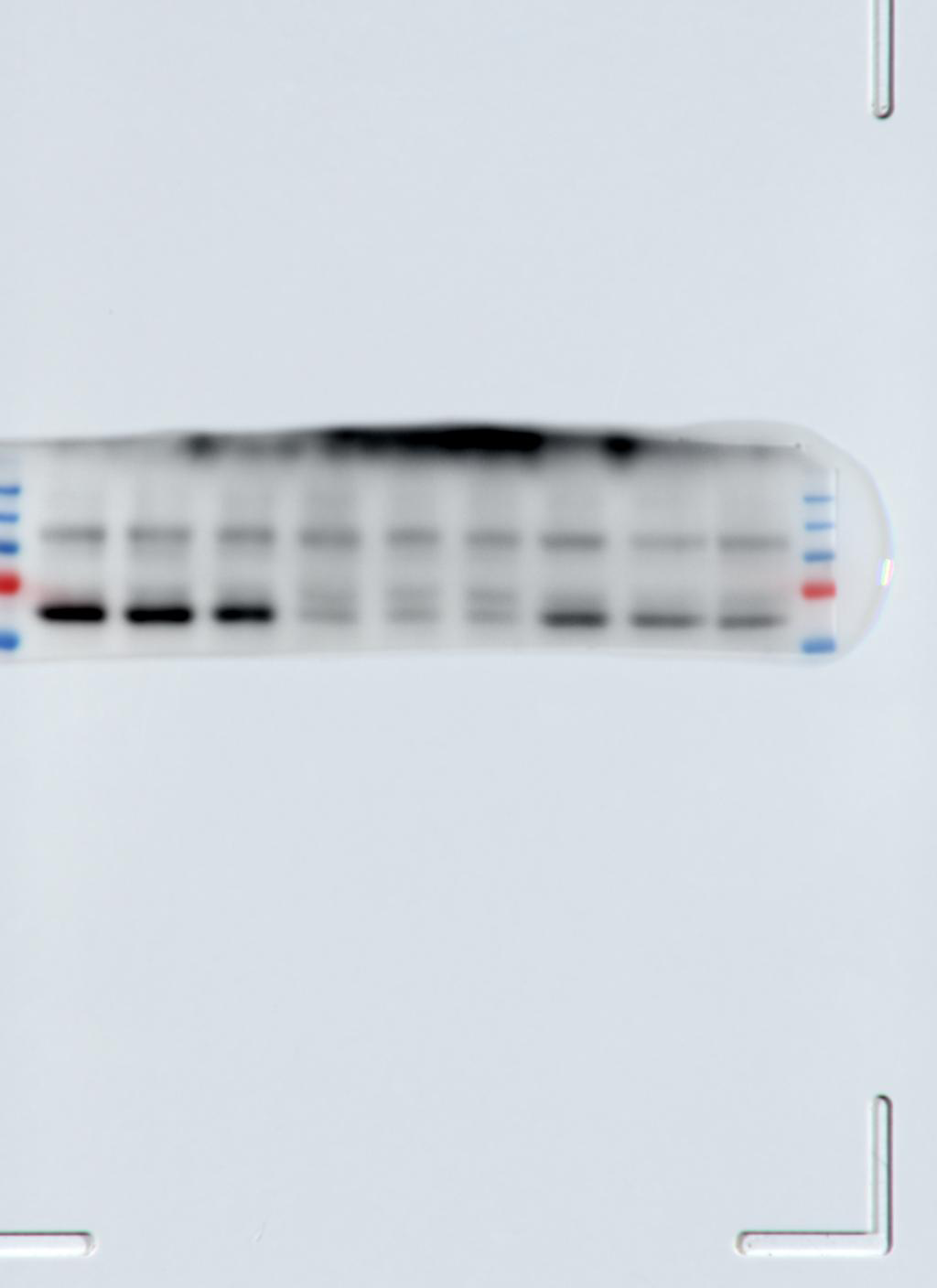

Supplement: Supplemental Information 1 [file peerj-10-13499-s001.zip › WB results of JB treating in hela-shWASH cells/WB results of JB treating in hela-shWASH cells-First/WASH/WASH.png]

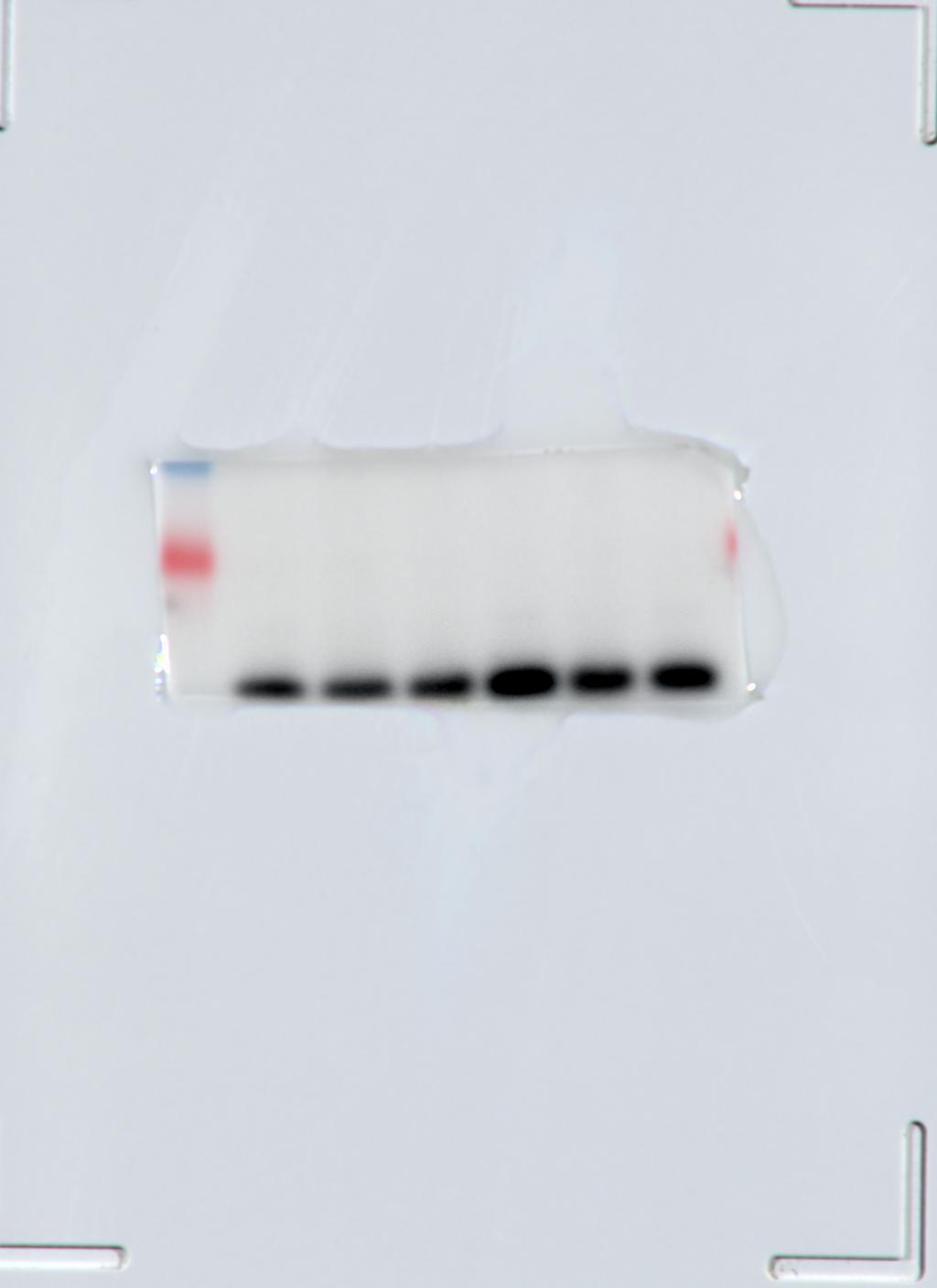

Supplement: Supplemental Information 1 [file peerj-10-13499-s001.zip › WB results of JB treating in hela-shWASH cells/WB results of JB treating in hela-shWASH cells-Second/BAX/BAX.png]

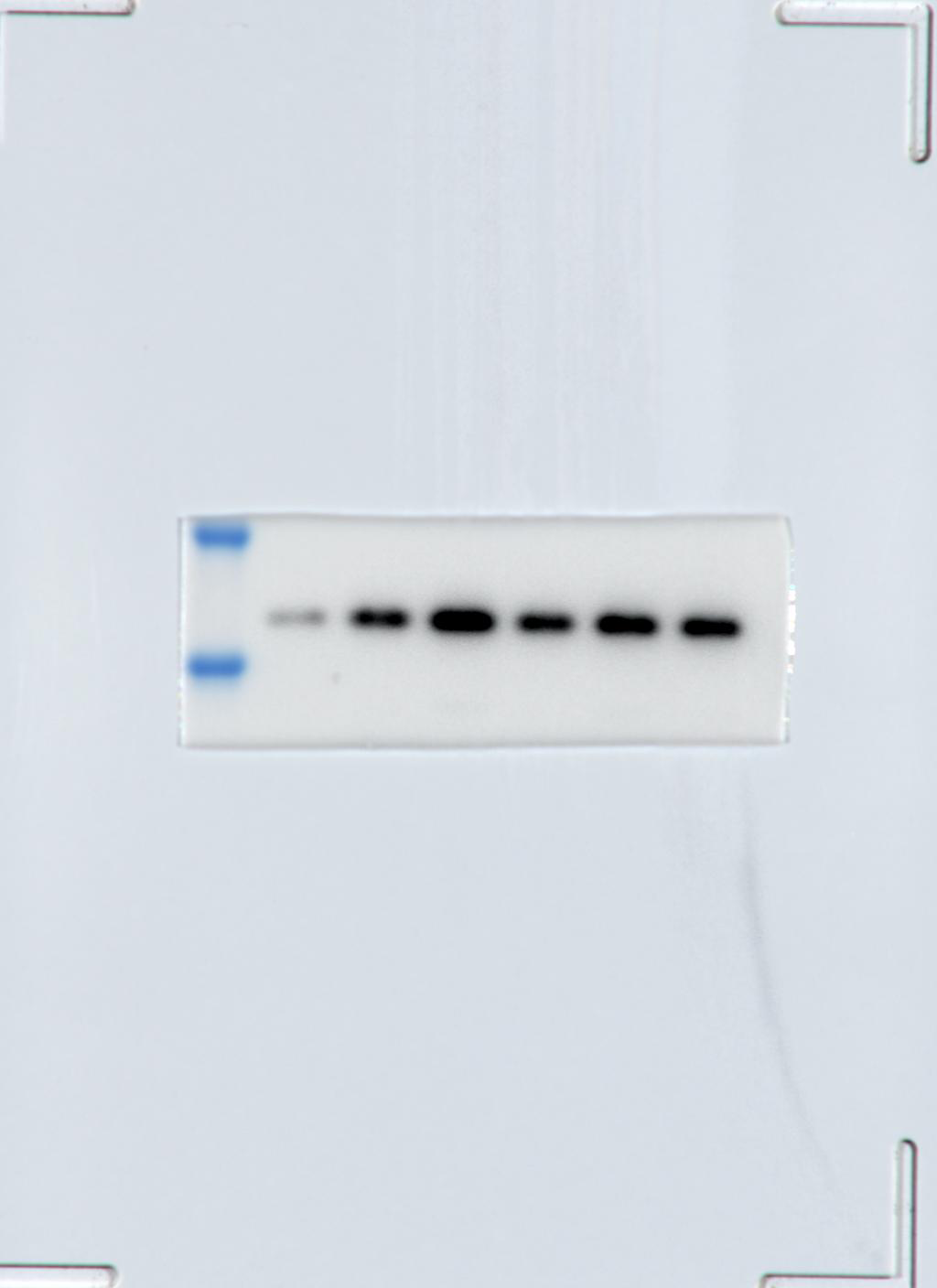

Supplement: Supplemental Information 1 [file peerj-10-13499-s001.zip › WB results of JB treating in hela-shWASH cells/WB results of JB treating in hela-shWASH cells-Second/BCL2/BCL.png]

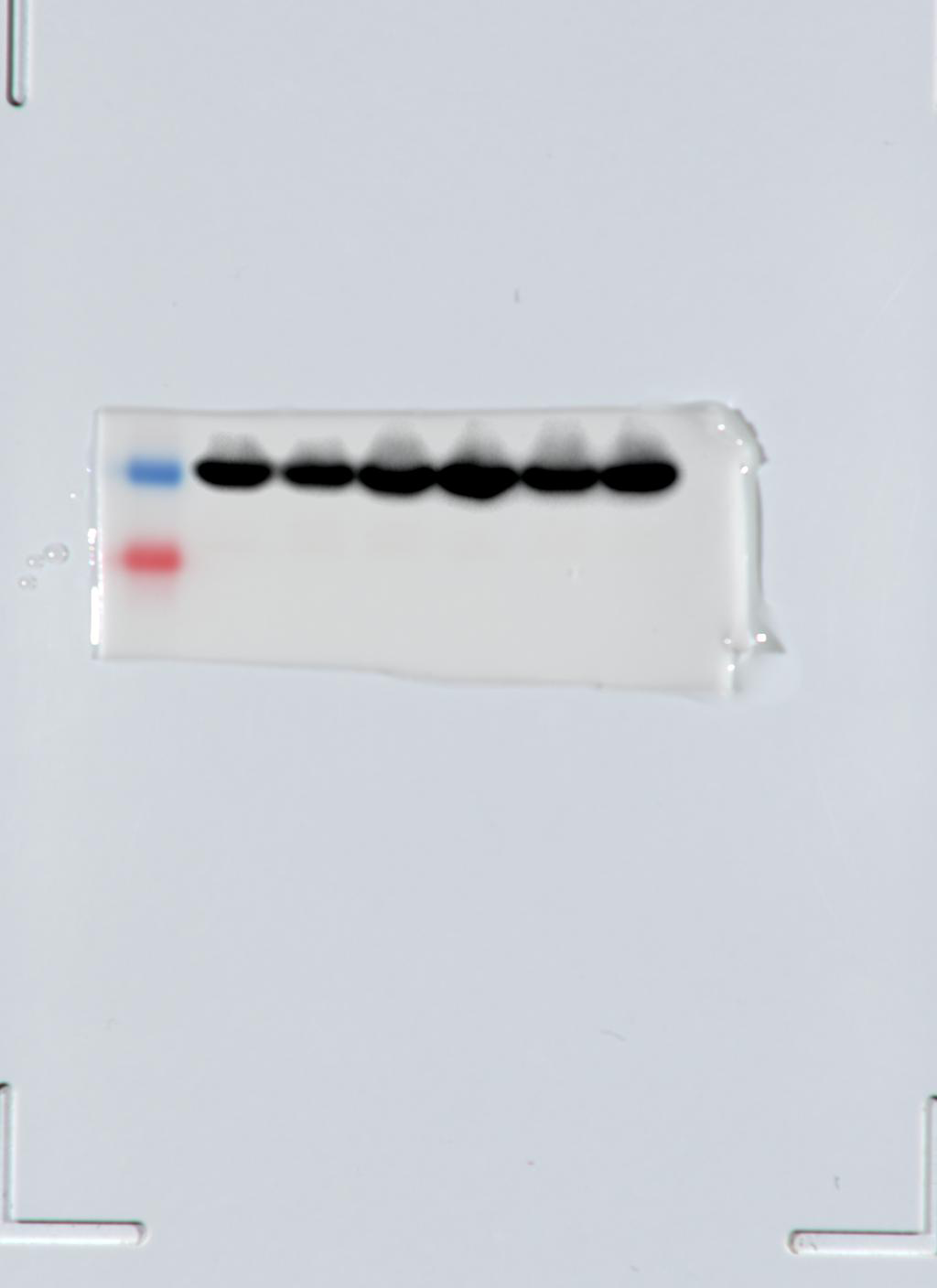

Supplement: Supplemental Information 1 [file peerj-10-13499-s001.zip › WB results of JB treating in hela-shWASH cells/WB results of JB treating in hela-shWASH cells-Second/GAPDH/GAPDH.png]

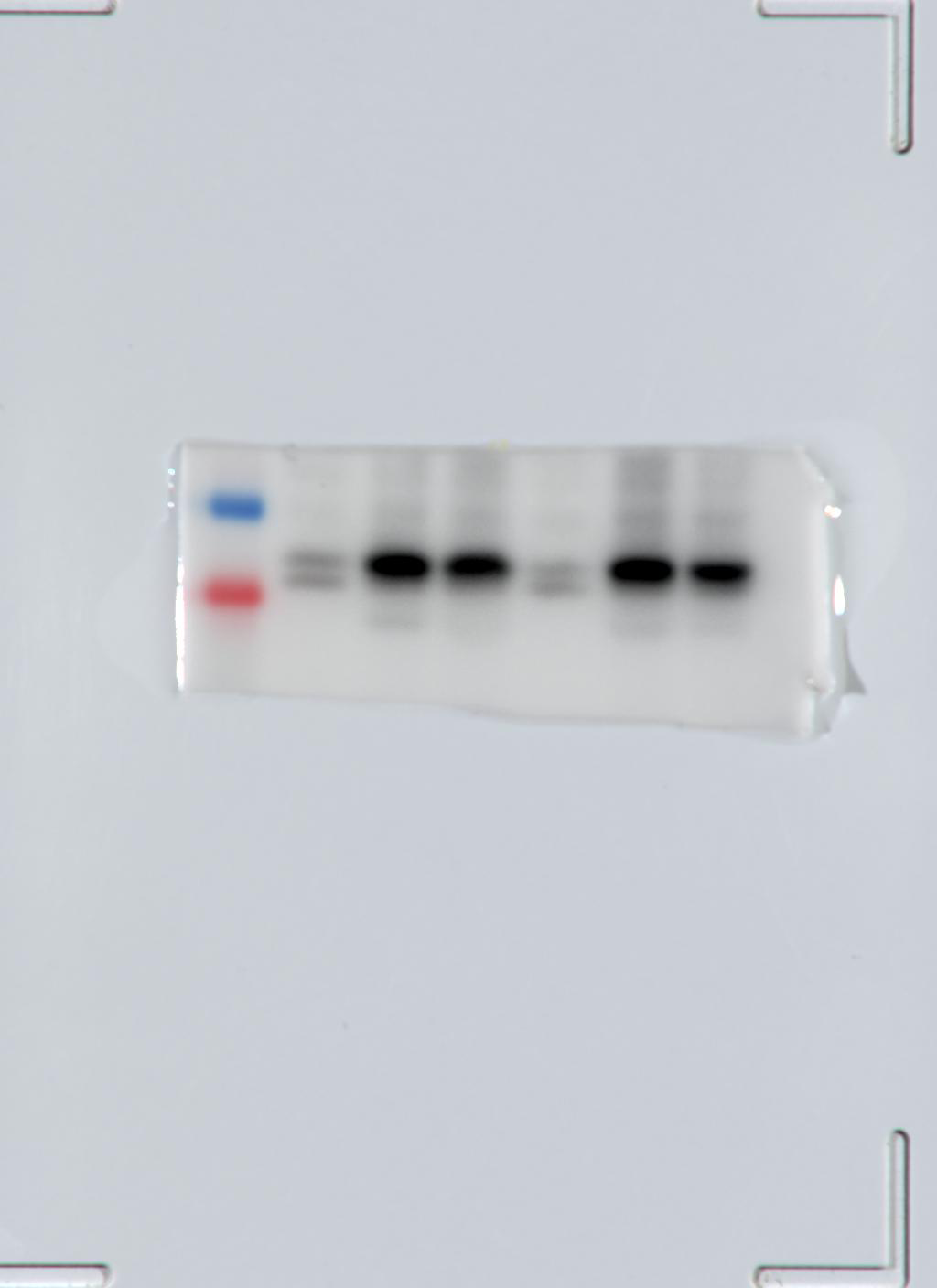

Supplement: Supplemental Information 1 [file peerj-10-13499-s001.zip › WB results of JB treating in hela-shWASH cells/WB results of JB treating in hela-shWASH cells-Second/HO-1/HO-1.png]

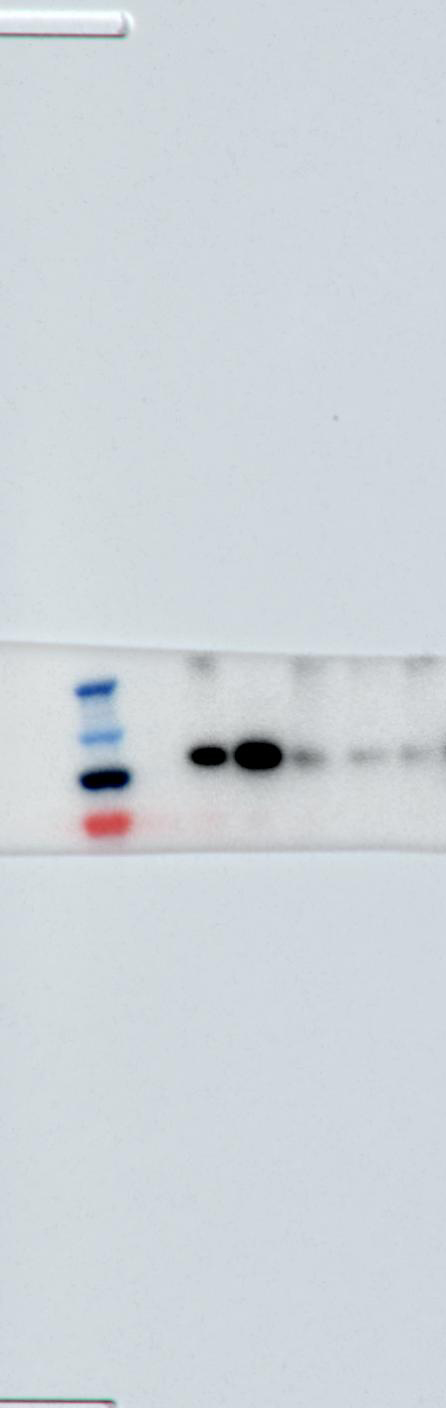

Supplement: Supplemental Information 1 [file peerj-10-13499-s001.zip › WB results of JB treating in hela-shWASH cells/WB results of JB treating in hela-shWASH cells-Second/NRF2/NRF2.png]

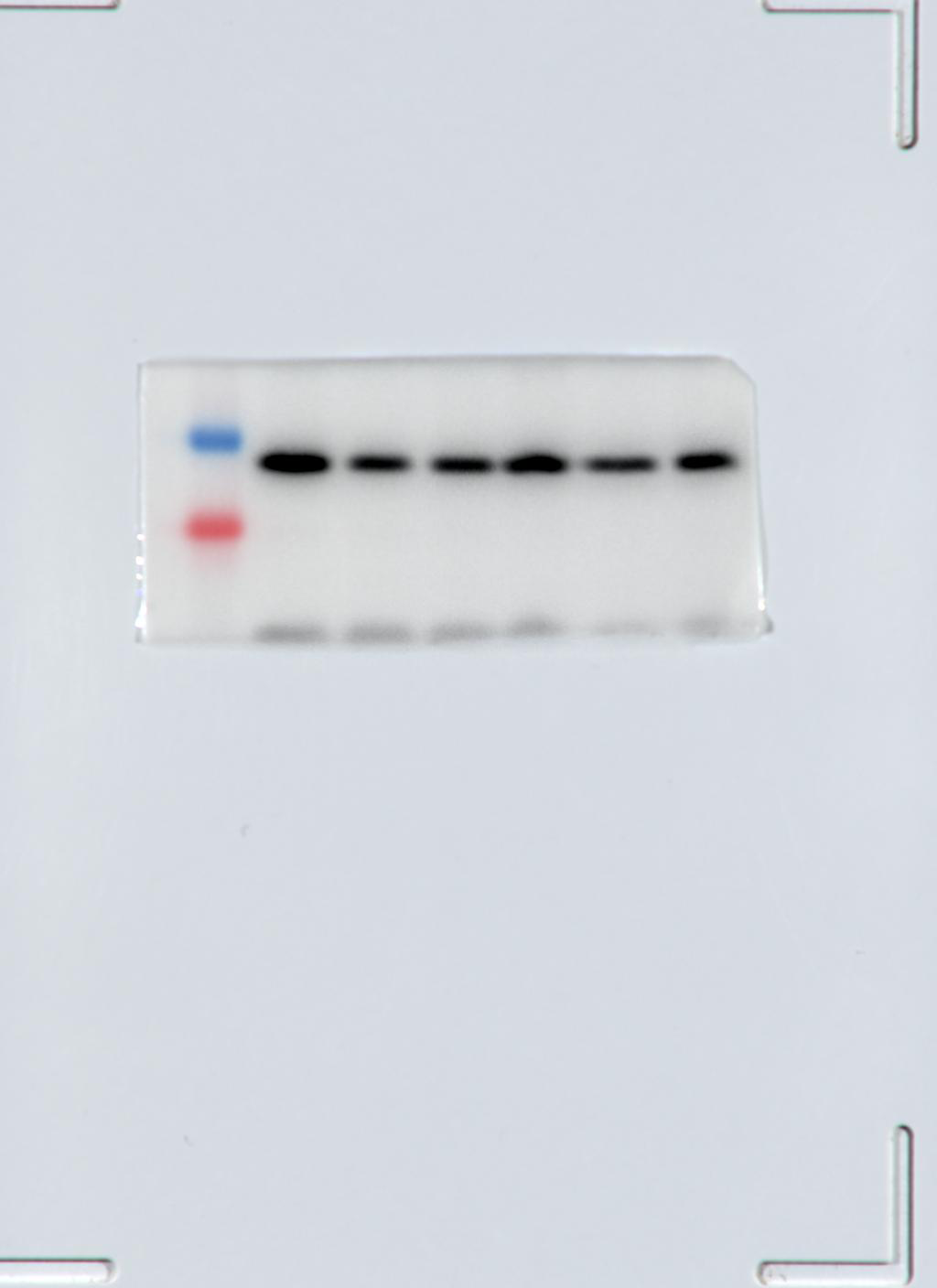

Supplement: Supplemental Information 1 [file peerj-10-13499-s001.zip › WB results of JB treating in hela-shWASH cells/WB results of JB treating in hela-shWASH cells-Second/PCNA/PCNA.png]

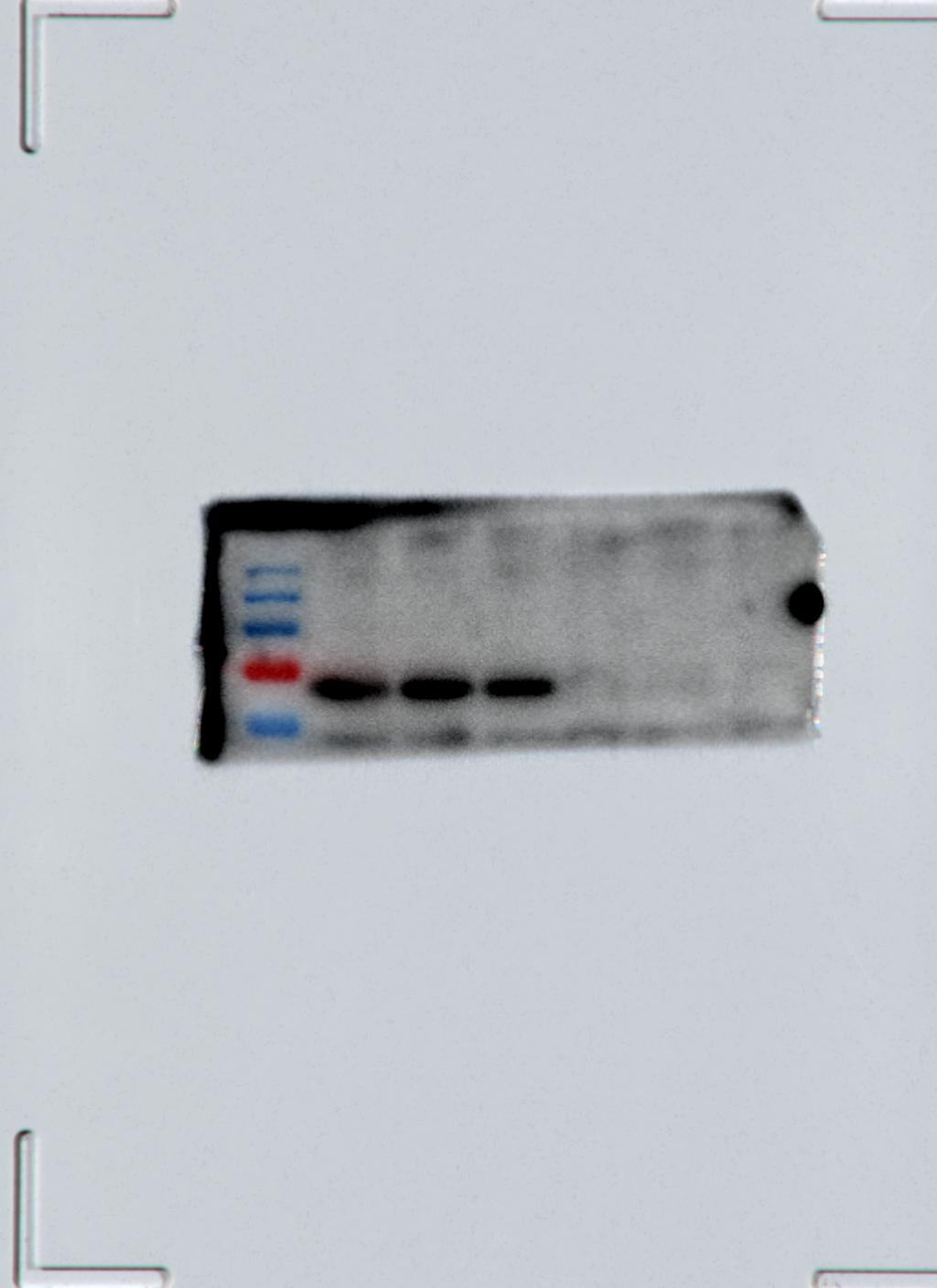

Supplement: Supplemental Information 1 [file peerj-10-13499-s001.zip › WB results of JB treating in hela-shWASH cells/WB results of JB treating in hela-shWASH cells-Second/WASH/WASH.png]

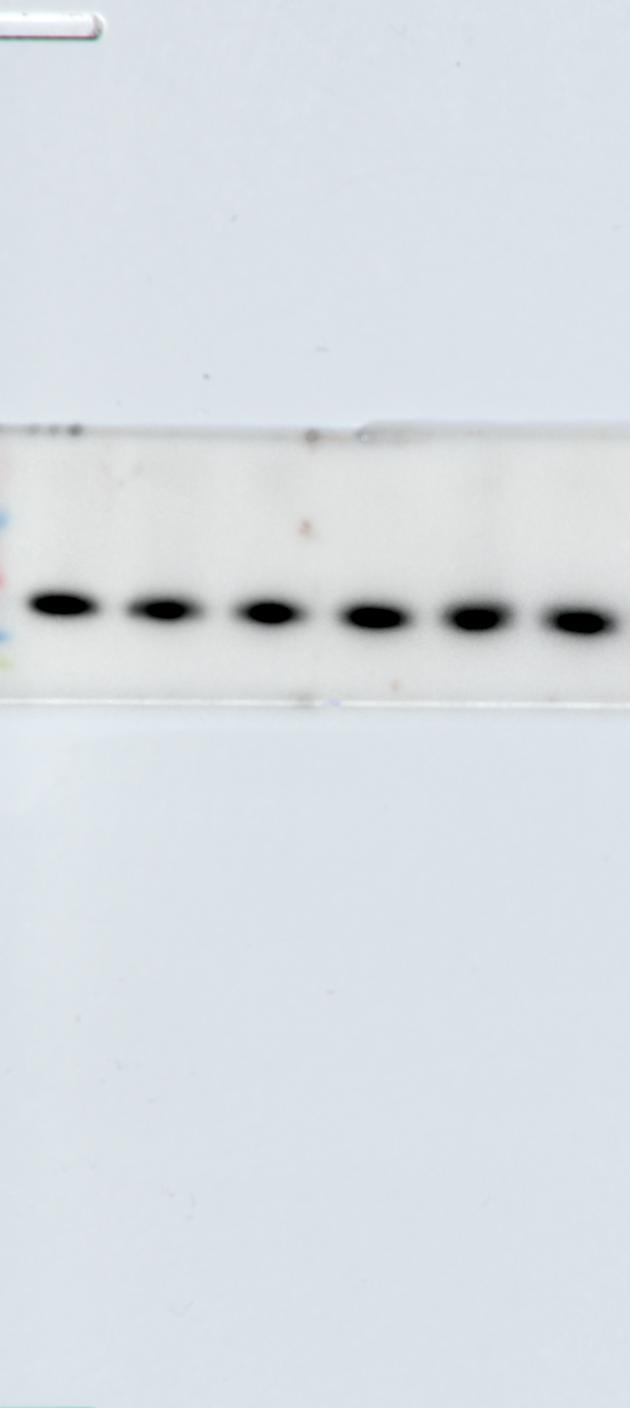

Supplement: Supplemental Information 1 [file peerj-10-13499-s001.zip › WB results of JB treating in hela-shWASH cells/WB results of JB treating in hela-shWASH cells-Third/BAX/BAX.png]

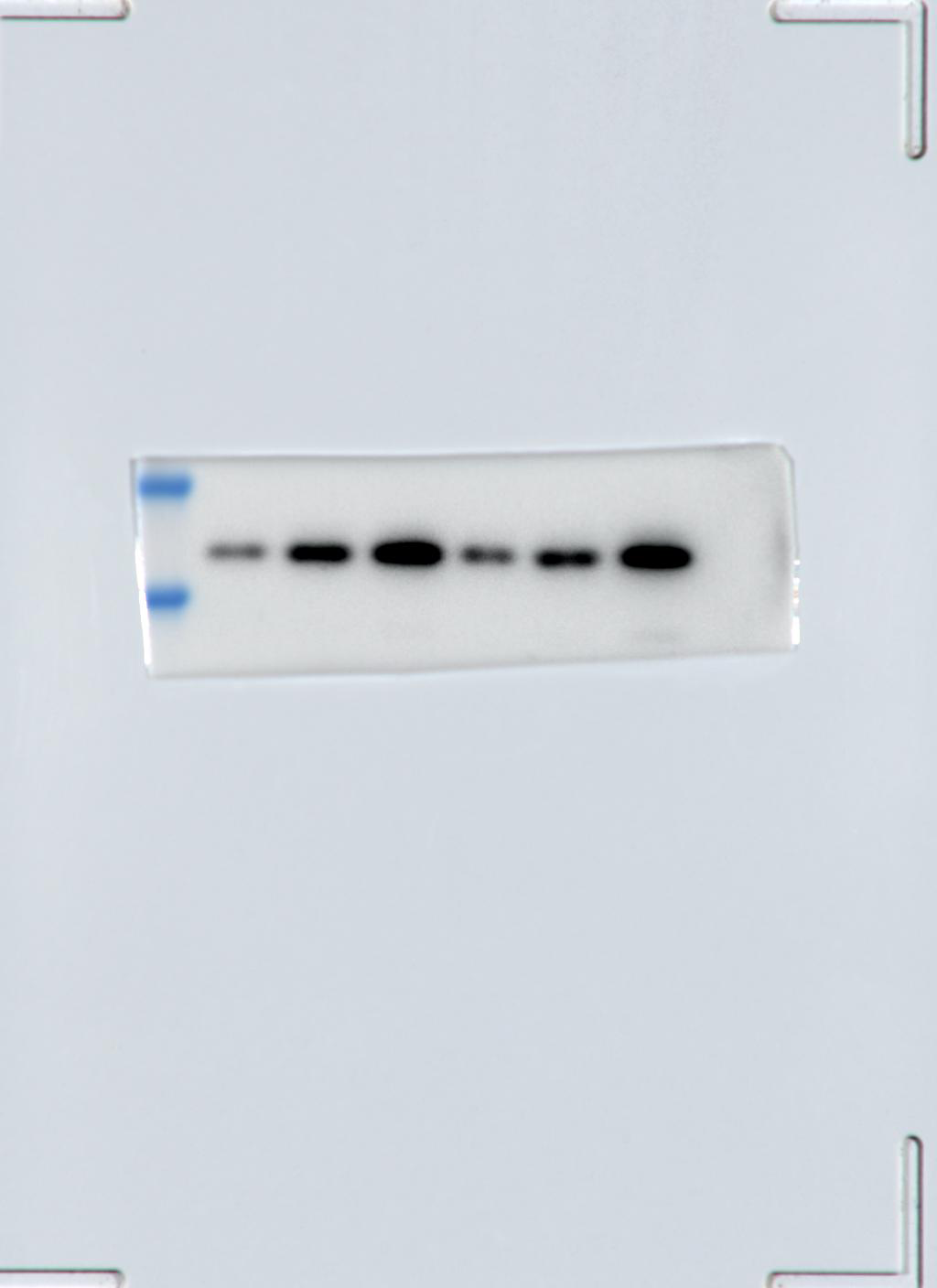

Supplement: Supplemental Information 1 [file peerj-10-13499-s001.zip › WB results of JB treating in hela-shWASH cells/WB results of JB treating in hela-shWASH cells-Third/BCL2/BCL.png]

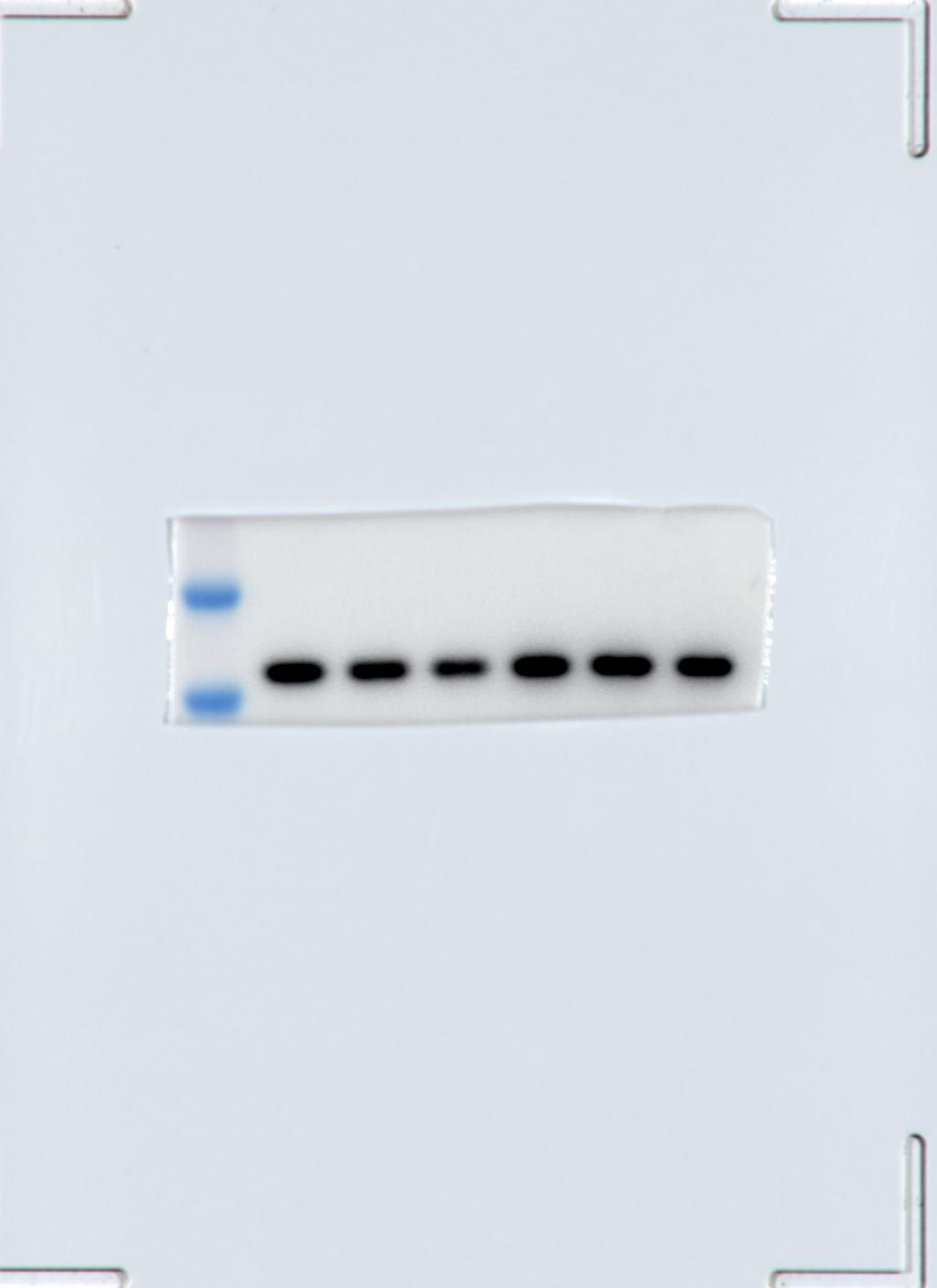

Supplement: Supplemental Information 1 [file peerj-10-13499-s001.zip › WB results of JB treating in hela-shWASH cells/WB results of JB treating in hela-shWASH cells-Third/GAPDH/GAPDH.png]

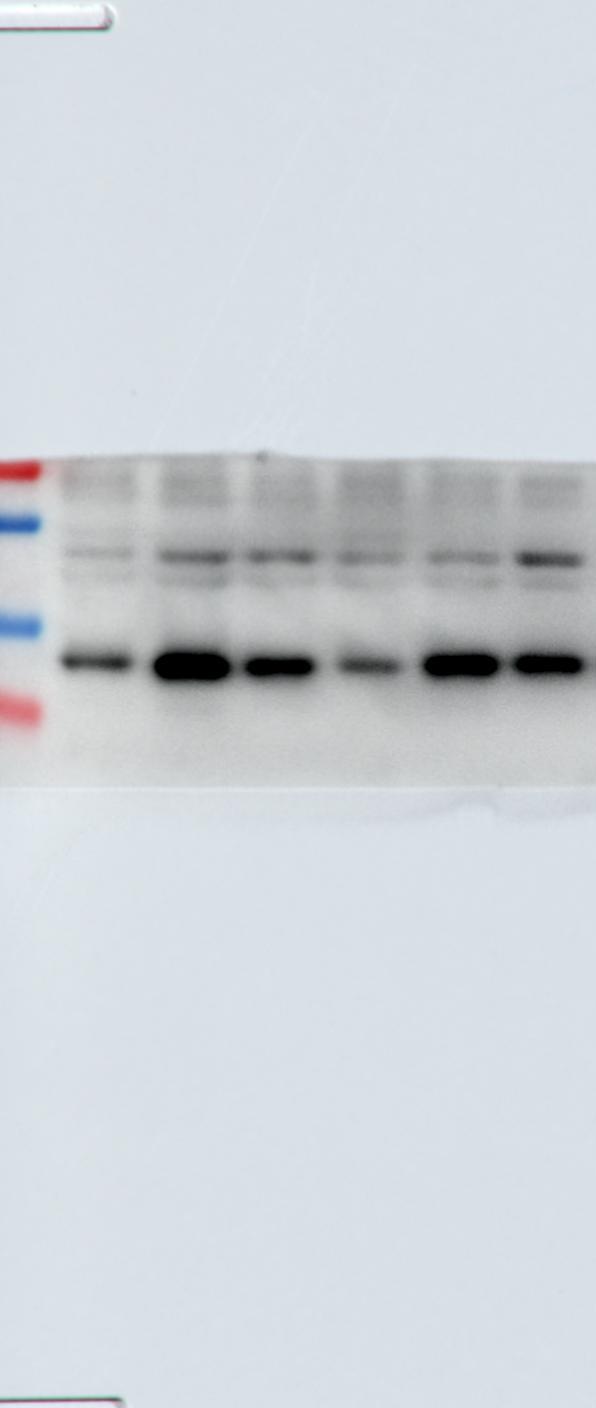

Supplement: Supplemental Information 1 [file peerj-10-13499-s001.zip › WB results of JB treating in hela-shWASH cells/WB results of JB treating in hela-shWASH cells-Third/HO-1/HO-1.png]

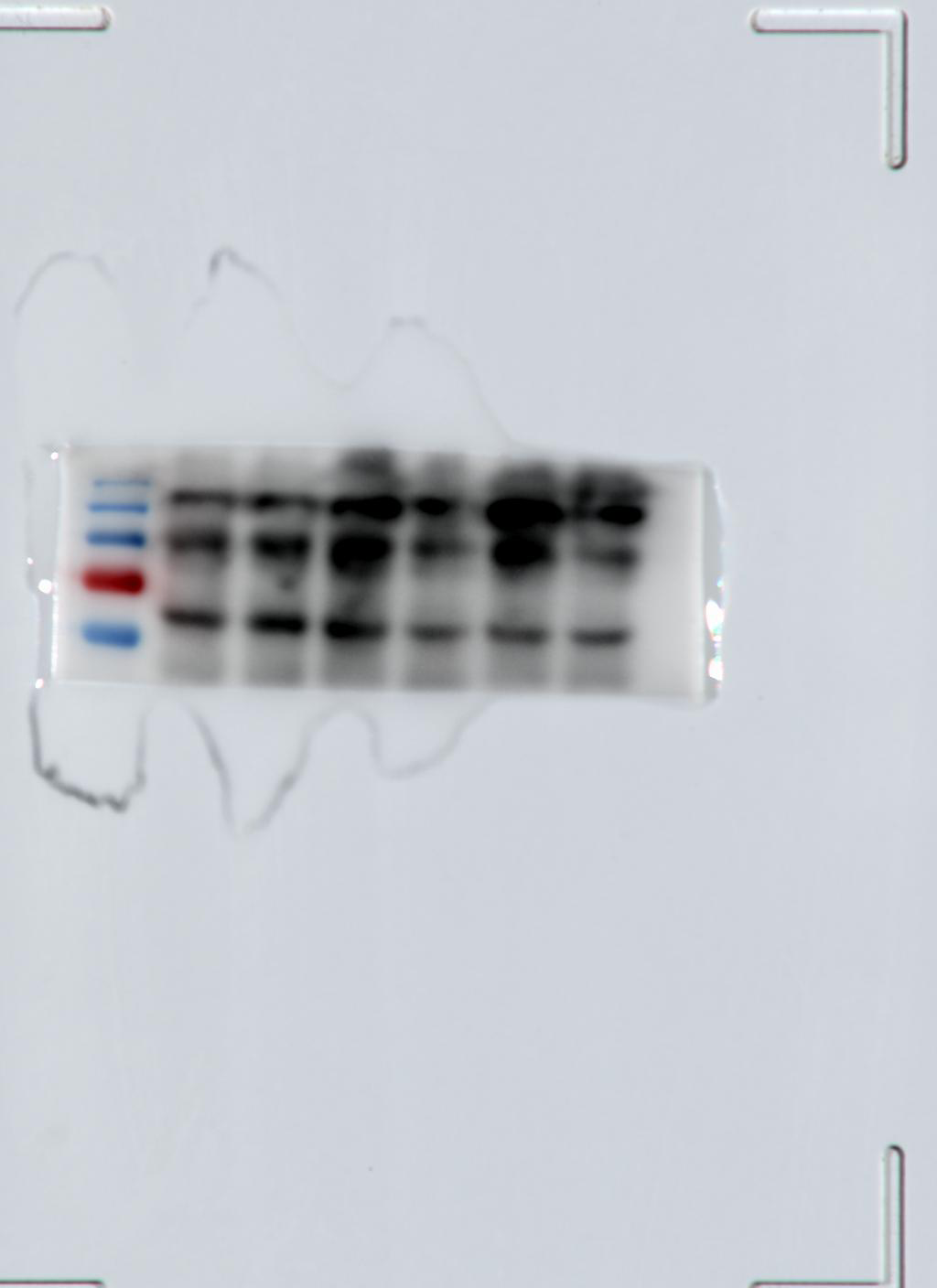

Supplement: Supplemental Information 1 [file peerj-10-13499-s001.zip › WB results of JB treating in hela-shWASH cells/WB results of JB treating in hela-shWASH cells-Third/NRF2/NRF2.png]

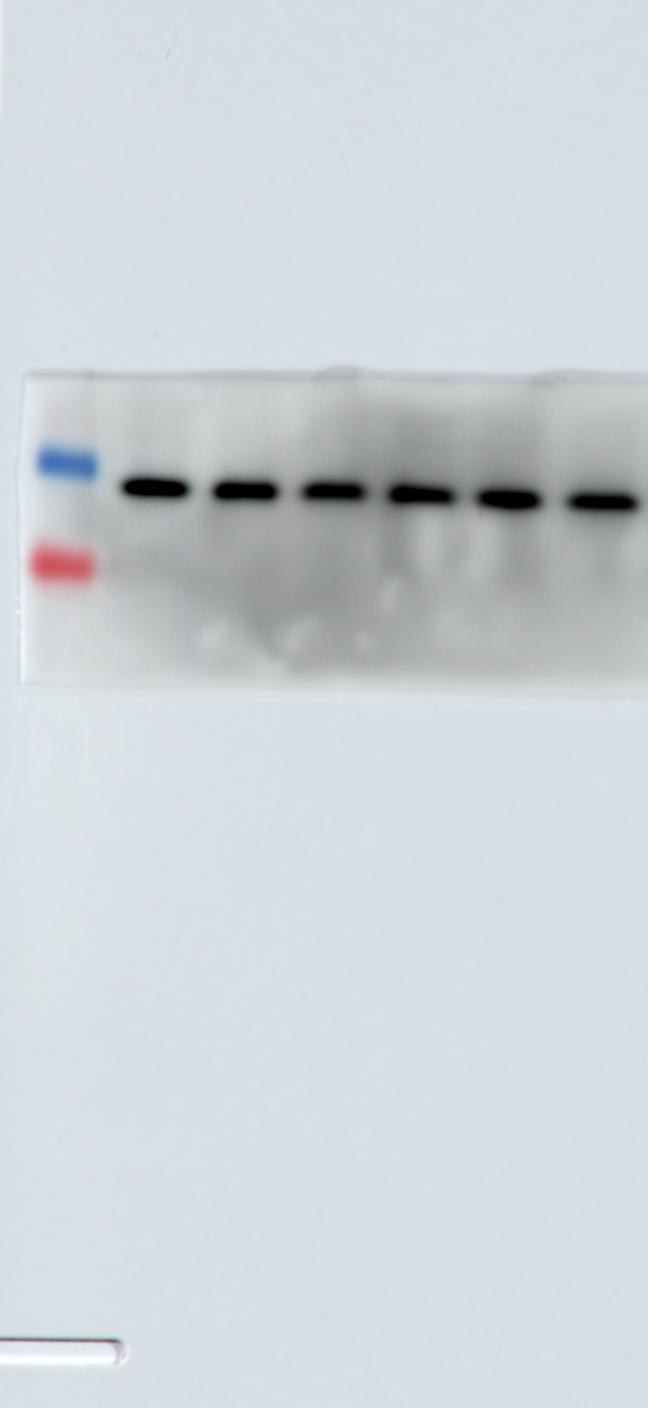

Supplement: Supplemental Information 1 [file peerj-10-13499-s001.zip › WB results of JB treating in hela-shWASH cells/WB results of JB treating in hela-shWASH cells-Third/PCNA/PCNA.png]

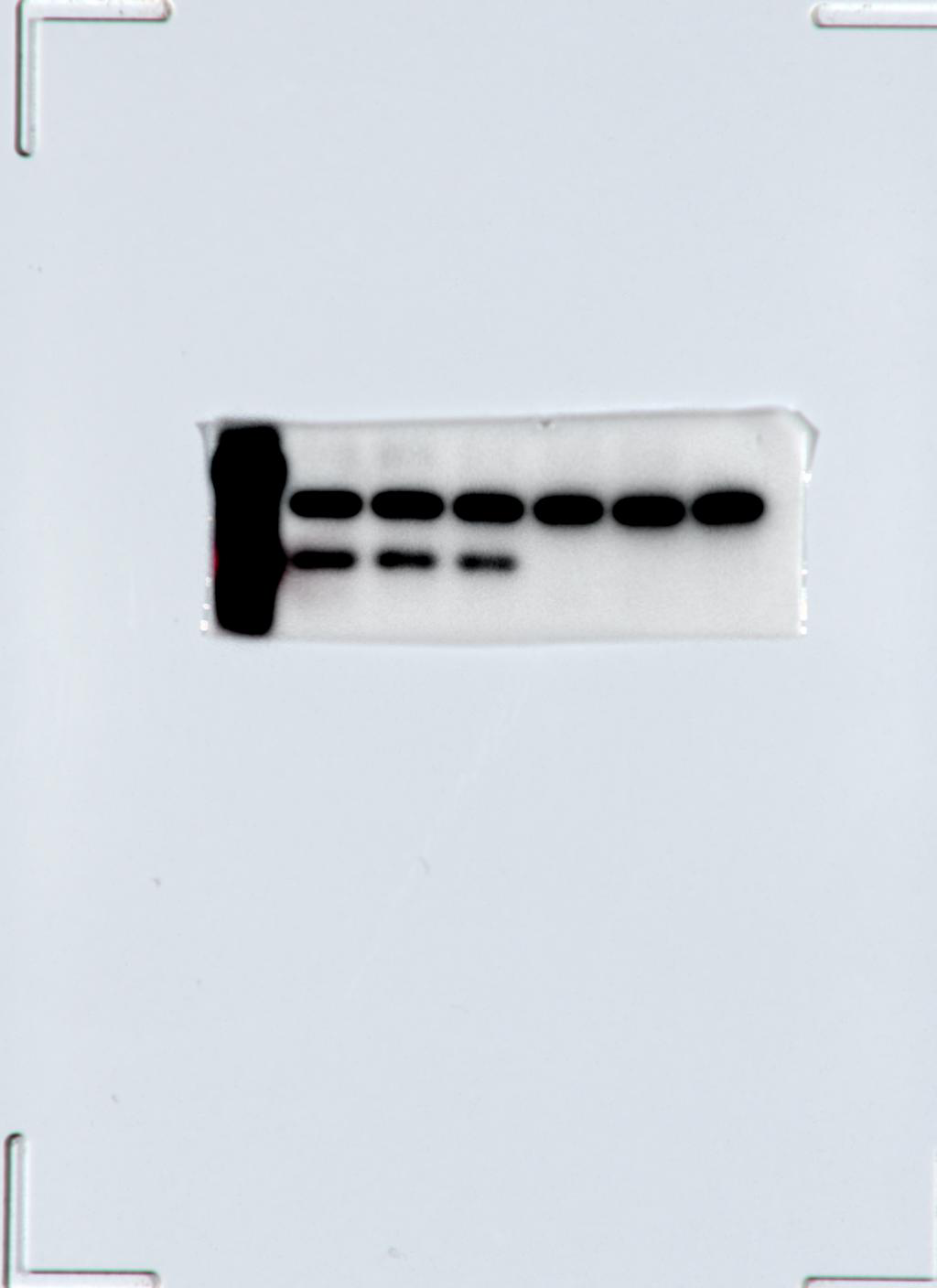

Supplement: Supplemental Information 1 [file peerj-10-13499-s001.zip › WB results of JB treating in hela-shWASH cells/WB results of JB treating in hela-shWASH cells-Third/WASH/WASH.png]
